# Qualitative and mixed methods in systematic reviews

**DOI:** 10.1186/s13643-015-0151-y

**Published:** 2015-12-15

**Authors:** David Gough

**Affiliations:** EPPI-Centre, Social Science Research Unit, University College London, London, WC1H 0NR UK

## Expanding the range of methods of systematic review

The logic of systematic reviews is very simple. We use transparent rigorous approaches to undertake primary research, and so we should do the same in bringing together studies to describe what has been studied (a research map) or to integrate the findings of the different studies to answer a research question (a research synthesis). We should not really need to use the term ‘systematic’ as it should be assumed that researchers are using and reporting systematic methods in all of their research, whether primary or secondary. Despite the universality of this logic, systematic reviews (maps and syntheses) are much better known in health research and for answering questions of the effectiveness of interventions (what works). Systematic reviews addressing other sorts of questions have been around for many years, as in, for example, meta ethnography [1] and other forms of conceptual synthesis [2], but only recently has there been a major increase in the use of systematic review approaches to answer other sorts of research questions.

There are probably several reasons for this broadening of approach. One may be that the increased awareness of systematic reviews has made people consider the possibilities for all areas of research. A second related factor may be that more training and funding resources have become available and increased the capacity to undertake such varied review work.

A third reason could be that some of the initial anxieties about systematic reviews have subsided. Initially, there were concerns that their use was being promoted by a new managerialism where reviews, particularly effectiveness reviews, were being used to promote particular ideological and theoretical assumptions and to indirectly control research agendas. However, others like me believe that explicit methods should be used to enable transparency of perspectives driving research and to open up access to and participation in research agendas and priority setting [3] as illustrated, for example, by the James Lind Alliance (see http://www.jla.nihr.ac.uk/).

A fourth possible reason for the development of new approaches is that effectiveness reviews have themselves broadened. Some ‘what works’ reviews can be open to criticism for only testing a ‘black box’ hypothesis of what works with little theorizing or any logic model about why any such hypothesis should be true and the mechanisms involved in such processes. There is now more concern to develop theory and to test how variables combine and interact. In primary research, qualitative strategies are advised prior to undertaking experimental trials [4, 5] and similar approaches are being advocated to address complexity in reviews [6], in order to ask questions and use methods that address theories and processes that enable an understanding of both impact and context.

This Special Issue of *Systematic Reviews Journal* is providing a focus for these new methods of review whether these use qualitative review methods on their own or mixed together with more quantitative approaches. We are linking together with the sister journal *Trials* for this Special Issue as there is a similar interest in what qualitative approaches can and should contribute to primary research using experimentally controlled trials (see *Trials* Special Issue editorial by Claire Snowdon).

## Dimensions of difference in reviews

Developing the range of methods to address different questions for review creates a challenge in describing and understanding such methods. There are many names and brands for the new methods which may or may not withstand the changes of historical time, but another way to comprehend the changes and new developments is to consider the dimensions on which the approaches to review differ [7, 8].

One important distinction is the research question being asked and the associated paradigm underlying the method used to address this question. Research assumes a particular theoretical position and then gathers data within this conceptual lens. In some cases, this is a very specific hypothesis that is then tested empirically, and sometimes, the research is more exploratory and iterative with concepts being emergent and constructed during the research process. This distinction is often labelled as quantitative or positivist versus qualitative or constructionist. However, this can be confusing as much research taking a ‘quantitative’ perspective does not have the necessary numeric data to analyse. Even if it does have such data, this might be explored for emergent properties. Similarly, research taking a ‘qualitative’ perspective may include implicit quantitative themes in terms of the extent of different qualitative findings reported by a study.

Sandelowski and colleagues’ solution is to consider the analytic activity and whether this aggregates (adds up) or configures (arranges) the data [9]. In a randomized controlled trial and an effectiveness review of such studies, the main analysis is the aggregation of data using a priori non-emergent strategies with little iteration. However, there may also be post hoc analysis that is more exploratory in arranging (configuring) data to identify patterns as in, for example, meta regression or qualitative comparative analysis aiming to identify the active ingredients of effective interventions [10]. Similarly, qualitative primary research or reviews of such research are predominantly exploring emergent patterns and developing concepts iteratively, yet there may be some aggregation of data to make statements of generalizations of extent.

Even where the analysis is predominantly configuration, there can be a wide variation in the dimensions of difference of iteration of theories and concepts. In thematic synthesis [11], there may be few presumptions about the concepts that will be configured. In meta ethnography which can be richer in theory, there may be theoretical assumptions underlying the review question framing the analysis. In framework synthesis, there is an explicit conceptual framework that is iteratively developed and changed through the review process [12, 13].

In addition to the variation in question, degree of configuration, complexity of theory, and iteration are many other dimensions of difference between reviews. Some of these differences follow on from the research questions being asked and the research paradigm being used such as in the approach to searching (exhaustive or based on exploration or saturation) and the appraisal of the quality and relevance of included studies (based more on risk of bias or more on meaning). Others include the extent that reviews have a broad question, depth of analysis, and the extent of resultant ‘work done’ in terms of progressing a field of inquiry [7, 8].

## Mixed methods reviews

As one reason for the growth in qualitative synthesis is what they can add to quantitative reviews, it is not surprising that there is also growing interest in mixed methods reviews. This reflects similar developments in primary research in mixing methods to examine the relationship between theory and empirical data which is of course the cornerstone of much research. But, both primary and secondary mixed methods research also face similar challenges in examining complex questions at different levels of analysis and of combining research findings investigated in different ways and may be based on very different epistemological assumptions [14, 15].

Some mixed methods approaches are convergent in that they integrate different data and methods of analysis together at the same time [16, 17]. Convergent systematic reviews could be described as having broad inclusion criteria (or two or more different sets of criteria) for methods of primary studies and have special methods for the synthesis of the resultant variation in data. Other reviews (and also primary mixed methods studies) are sequences of sub-reviews in that one sub-study using one research paradigm is followed by another sub-study with a different research paradigm. In other words, a qualitative synthesis might be used to explore the findings of a prior quantitative synthesis or vice versa [16, 17].

An example of a predominantly aggregative sub-review followed by a configuring sub-review is the EPPI-Centre’s mixed methods review of barriers to healthy eating [18]. A sub-review on the effectiveness of public health interventions showed a modest effect size. A configuring review of studies of children and young people’s understanding and views about eating provided evidence that the public health interventions did not take good account of such user views research, and that the interventions most closely aligned to the user views were the most effective. The already mentioned qualitative comparative analysis to identify the active ingredients within interventions leading to impact could also be considered a qualitative configuring investigation of an existing quantitative aggregative review [10].

An example of a predominantly configurative review followed by an aggregative review is realist synthesis. Realist reviews examine the evidence in support of mid-range theories [19] with a first stage of a configuring review of what is proposed by the theory or proposal (what would need to be in place and what casual pathways would have to be effective for the outcomes proposed by the theory to be supported?) and a second stage searching for empirical evidence to test for those necessary conditions and effectiveness of the pathways. The empirical testing does not however use a standard ‘what works’ a priori methods approach but rather a more iterative seeking out of evidence that confirms or undermines the theory being evaluated [20].

Although sequential mixed methods approaches are considered to be sub-parts of one larger study, they could be separate studies as part of a long-term strategic approach to studying an issue. We tend to see both primary studies and reviews as one-off events, yet reviews are a way of examining what we know and what more we want to know as a strategic approach to studying an issue over time. If we are in favour of mixing paradigms of research to enable multiple levels and perspectives and mixing of theory development and empirical evaluation, then we are really seeking mixed methods research strategies rather than simply mixed methods studies and reviews.
